# In Situ *N*-Glycosylation Signatures of Epithelial Ovarian Cancer Tissue as Defined by MALDI Mass Spectrometry Imaging

**DOI:** 10.3390/cancers14041021

**Published:** 2022-02-17

**Authors:** Marta Grzeski, Eliane T. Taube, Elena I. Braicu, Jalid Sehouli, Véronique Blanchard, Oliver Klein

**Affiliations:** 1 Institute of Diagnostic Laboratory Medicine, Clinical Chemistry and Pathobiochemistry, Charité—Universitätsmedizin Berlin, Corporate Member of Freie Universität Berlin, Humboldt-Universität zu Berlin, and Berlin Institute of Health, 13353 Berlin, Germany; marta.grzeski@charite.de; 2 Institute of Pathology, Charité—Universitätsmedizin Berlin, Corporate Member of Freie Universität Berlin, Humboldt-Universität zu Berlin, and Berlin Institute of Health, 10117 Berlin, Germany; eliane.taube@charite.de; 3 Department of Gynecology, European Competence Center for Ovarian Cancer, Charité—Universitätsmedizin Berlin, Corporate Member of Freie Universität Berlin, Humboldt-Universität zu Berlin, and Berlin Institute of Health, 13353 Berlin, Germany; elena.braicu@charite.de (E.I.B.); jalid.sehouli@charite.de (J.S.); 4 BIH Center for Regenerative Therapies, Charité—Universitätsmedizin Berlin, Corporate Member of Freie Universität Berlin, Humboldt-Universität zu Berlin, and Berlin Institute of Health, 13353 Berlin, Germany; oliver.klein@charite.de

**Keywords:** in situ *N*-glycosylation, sialylation, ovarian cancer, EOC, imaging, MALDI-MSI

## Abstract

**Simple Summary:**

Altered glycosylation of proteins was shown to be implicated in various steps of malignant transformation and tumor progression. For that reason, it is frequently referred to as the next hallmark of cancer. In epithelial ovarian cancer (EOC), a female gynecological malignancy of the highest mortality, *N*-glycosylation alterations were predominantly investigated at the level of serum glycoproteins and OC cell lines. By contrast, data on in situ *N*-glycosylation in OC tissue are still very limited, particularly with respect to terminal sialylation. This is despite the increasing number of studies supporting the role of sialylated *N*-glycans in tumor progression, angiogenesis, and metastasis. In this work, MALDI mass spectrometry imaging (MALDI-MSI) was implemented in combination with chemical sialic acid derivatization to determine tissue type-specific *N*-glycosylation of less common histotypes of EOC and non-malignant ovarian disease.

**Abstract:**

The particularly high mortality of epithelial ovarian cancer (EOC) is in part linked to limited understanding of its molecular signatures. Although there are data available on in situ *N*-glycosylation in EOC tissue, previous studies focused primarily on neutral *N*-glycan species and, hence, still little is known regarding EOC tissue-specific sialylation. In this proof-of-concept study, we implemented MALDI mass spectrometry imaging (MALDI-MSI) in combination with sialic acid derivatization to simultaneously investigate neutral and sialylated *N*-glycans in formalin-fixed paraffin-embedded tissue microarray specimens of less common EOC histotypes and non-malignant borderline ovarian tumor (BOT). The applied protocol allowed detecting over 50 *m/z* species, many of which showed differential tissue distribution. Most importantly, it could be demonstrated that α2,6- and α2,3-sialylated *N*-glycans are enriched in tissue regions corresponding to tumor and adjacent tumor-stroma, respectively. Interestingly, analogous *N*-glycosylation patterns were observed in tissue cores of BOT, suggesting that regio-specific *N*-glycan distribution might occur already in non-malignant ovarian pathologies. All in all, our data provide proof that the combination of MALDI-MSI and sialic acid derivatization is suitable for delineating regio-specific *N*-glycan distribution in EOC and BOT tissues and might serve as a promising strategy for future glycosylation-based biomarker discovery studies.

## 1. Introduction

According to recent statistics, each year approximately 300,000 women worldwide are diagnosed with ovarian cancer (OC) and 180,000 succumb to this disease. Thus, even though OC is rather rare as compared to other malignancies, it is the fifth most common cause of female cancer death and the leading cause of cancer death among gynecological malignancies in women [1,2]. Primarily, this high mortality is due to inadequate early detection that results from vague, late-occurring disease symptoms and a lack of accurate early diagnostic markers. Thus, in the absence of protein-based biomarkers allowing OC detection at an early, still curable stage, altered glycosylation of proteins attracted attention as a promising source of novel complementary screening markers [3].

OC is far from being a single disease as it has many different etiological and histological subtypes. The most common epithelial ovarian cancer (EOC) accounts for about 90% of all ovarian malignancies and is categorized into five histotypes, i.e., high-grade serous, low-grade serous, clear cell, endometrioid, and mucinous [4]. Of these, high-grade serous ovarian carcinoma (HGSOC) is the most common and aggressive, whereas low-grade serous (LGSOC), clear cell (CCC), endometrioid (EC), and mucinous ovarian carcinomas, which altogether correspond to ~30% of all OC cases, are typically characterized by less aggressive behaviors [4,5]. Due to their lower incidence, these ovarian carcinomas are also less frequently studied as compared to the most common high-grade serous histotype.

Among different glycosylation types, asparagine-linked glycosylation, regularly referred to as *N*-glycosylation, is the most commonly studied in the context of cancer biomarkers [6,7]. This is largely owed to the ubiquity and the fundamental importance of *N*-glycans in various biological processes (e.g., cell–cell and cell–matrix interactions, receptor binding, signal transduction, immune responses). Yet, while OC-associated *N*-glycosylation alterations were broadly investigated at the level of total and individual serum proteins [8,9,10,11,12], much less is known about alterations occurring directly in malignant ovarian tissues.

Until recently, the in situ visualization of *N*-glycans and other tissue-contained carbohydrates was only possible with the help of specific carbohydrate-binding proteins, e.g., lectins and anti-carbohydrate antibodies [13]. As far as this methodology has been routinely applied in the clinic (e.g., measurement of CA 19-9 in the prognosis of pancreatic cancer [14]), the anti-carbohydrate antibody- and lectin-based visualization is a highly targeted approach, in which investigated epitopes must be known and defined prior to the analysis. Additionally, as both lectins and anti-carbohydrate antibodies recognize structural carbohydrate motifs composed of 1–4 monosaccharides, they lack specificity toward glycan class and carrier. As such, their use does not typically allow determining whether the detected glycans stem from glycolipids or *N*- or *O*-linked glycoproteins [15]. Due to these constraints, *N*-glycosylation profiling is most commonly performed using tissue homogenates, which allows for more detailed structural analyses but lacks site specificity. Additionally, it generates information that cannot be directly correlated to tissue morphology, which is a serious drawback in cancer research.

Importantly, the above-described limitations can be readily circumvented by using matrix-assisted laser desorption/ionization mass spectrometry imaging (MALDI-MSI), a cutting-edge technique that combines high sensitivity of mass spectrometry with spatial distribution information, typical of immuno-histochemistry stainings. Following its successful employment in proteomics, lipidomics, and metabolomics [16,17,18], MALDI-MSI slowly paved its way to glycomics, where it is being used in an increasing number of applications, with tumor tissue analysis ranking high on the list [19,20,21,22].

To the best of our knowledge, there have been so far only two studies, in which the MALDI-MSI technique was employed to determine in situ *N*-glycosylation in OC tissues [20,22]. In both of them, investigated material consisted of tissue specimens of the most common serous histotype. In general, these studies provided reliable information regarding high-mannose- and hybrid-type *N*-glycans and proposed some *N*-glycan-based discriminants of distinct OC tissue regions. However, since positive ion mode MALDI-MSI was used in both of these studies without stabilizing labile sialic acid residues, the obtained *N*-glycome profiles might suffer from an inadequate detection of sialylated *N*-glycan species. It is important to note that, due to their terminal position, sialylated *N*-glycans play a particularly important role in tissue homeostasis and pathology. In fact, hypersialylation has long been associated with enhanced cancer cell survival and metastatic behavior [23,24]. For this reason, reliable detection of sialylated *N*-glycan species is essential for a comprehensive understanding of molecular changes accompanying the malignant transformation of ovarian tissues.

Therefore, in this proof-of-concept study, MALDI-MSI analysis of EOC tissue-specific *N*-glycosylation was further extended by implementing chemical derivatization of sialic acid residues, adapted from the protocol reported previously by Holst et. al. [21]. This approach allowed for improved and simultaneous detection of both neutral and sialylated *N*-glycan structures. To address the high complexity and heterogeneity of the disease, the investigated material consisted of formalin-fixed paraffin-embedded (FFPE) tissue microarray (TMA) specimens of the three less common EOC histotypes, namely LGSOC, CCC, and EC, for which *N*-glycosylation signatures have not yet been studied in situ. Additionally, to determine whether *N*-glycosylation patterns observed in EOC are as well represented in non-malignant ovarian pathologies, analogous analyses were performed using tissue specimens of borderline ovarian tumor (BOT). All in all, the aim of this study was to determine whether MALDI-MSI in combination with chemical derivatization of sialic acids [21] is suitable for the analysis of in situ *N*-glycosylation in EOC TMA specimens and whether EOC tissue *N*-glycans, particularly those terminated with α2,6- and α2,3-linked sialic acids, show regio-specific tissue distribution.

## 2. Materials and Methods

### 2.1. Chemicals and Reagents

Xylene, ethanol, trifluoroacetic acid (TFA), and ammonium hydroxide 25% were purchased from Merck (Darmstadt, Germany). Dimethylamine 40% (DMA), 1-Hydroxybenzotriazole hydrate (HOBt), Mayer’s hematoxylin solution, and eosin Y were from Sigma-Aldrich (Taufkirchen, Germany). Anhydrous dimethyl sulfoxide (DMSO) was from Applichem (Darmstadt, Germany). The 1-Ethyl-3-(3-dimethylaminopropyl) carbodiimide (EDC) was from Fluorochem (Hadfield, UK). Acetonitrile (ACN) was from VWR Chemicals (Darmstadt, Germany). Citric acid was from Carl Roth (Karlsruhe, Germany). The α-2,4-hydroxycinammic acid (CHCA) was from Bruker Daltonics (Bremen, Germany). PNGase F PRIME was from N-Zyme Scientifics (Doylestown, PA, USA). Dextran hydrolysate (DH) was prepared in-house from dextran (Oxford, UK). Milli-Q grade water was generated in-house using a Water purification system, MilliQ Plus (Merck Millipore, Darmstadt, Germany).

### 2.2. Tissue Sample Collection and Preparation

All tissue specimens were collected intraoperatively at the Department of Gynecology, Charité—Universitätsmedizin Berlin. Written, informed consent was obtained from all patients. The study was ethically approved by the Charité Institutional Ethics Committee (ethical vote number 207/2003). EOC tissue histotyping was performed by an experienced pathologist at the Institute of Pathology, Charité—Universitätsmedizin Berlin. All surgical specimens were formalin-fixed and paraffin-embedded (FFPE) according to standard protocols. The assembly of the tissue microarray (TMA) specimens was performed at the Institute of Pathology, Charité—Universitätsmedizin Berlin. The investigated material included four TMA slides, each of which consisted of tissue cores of different EOC histotype, namely LGSOC, CCC, EC, or non-malignant BOT (tissue cores included in the analysis are shown in Supplementary Material Figure S1). Five-μm-thick TMA sections were water bath-mounted on indium-tin-oxide (ITO)-coated glass slides (Bruker Daltonics, Bremen, Germany). After overnight drying at room temperature, tissue slides were stored protected from light at 4 °C until further use.

### 2.3. Deparaffinization, Rehydration, and Antigen Retrieval of EOC Tissues

All FFPE TMA slides were heat-treated at 60 °C for 1 h, after which they were dewaxed and rehydrated by washing in 100% xylene (1 × 5 min, 1 × 10 min), 100% ethanol (2 × 2 min), and MilliQ water (2 × 5 min). Rehydrated tissue slides were dried and stored overnight at room temperature in a vacuum desiccator. The following day, all tissues were antigen retrieved. The slides were incubated for 10 min in a near-boiling citric acid solution (98 °C, 10 mM, pH 6.0) and then were incubated for 30 min at 98 °C on a heating plate. The antigen retrieved slides were immersed in MilliQ water for 2 min and dried in a vacuum desiccator.

### 2.4. Chemical Derivatization of Sialic Acids

Negatively charged sialic acids were derivatized according to a protocol adapted from Holst et al. [21]. Briefly, dried tissue slides were incubated in 4 mL of DMSO containing 250 mM EDC, 500 mM HOBt, and 250 mM DMA for 2 h, 15 min at 60 °C in a moist chamber in an oven. This was followed by the addition of 1.6 mL of 25% ammonium hydroxide and a further incubation for 2 h at 60 °C. Upon completion of the reaction, tissue slides were rinsed thoroughly with 100% ethanol and then were sequentially immersed in 100% ethanol (2 × 2 min) and MilliQ water (2 × 5 min). Eventually, all tissues were dried in a vacuum desiccator.

### 2.5. In Situ PNGase F Deposition and Digestion

The PNGase F enzyme (0.12 μg/μL; buffer changed to MilliQ water using Amicon Ultra 0.5-mL centrifugal filters 10 kDa; Merck, Darmstadt, Germany) was deposited homogenously on each tissue using an automated spraying device (HTX TM-Sprayer; HTX Technologies, LLC, Chapel Hill, NC, USA), with the spraying parameters set as follows: temperature, 30 °C; flow rate, 0.015 mL/min; passes, 15; pattern, criss-cross; velocity, 750 mm/min; track spacing, 2 mm; gas flow rate, 2 L/min; pressure, 10 psi; drying time, 0 s; and nozzle height, 40 mm. The localization of the tissue within the glass slide (X and Y coordinates) was specified, allowing a 5-mm additional edge for the spray head to turn around off the tissue. For a negative control, a part of the tissue section or a whole tissue specimen was covered with a glass coverslip and secured with adhesive tape. Following the PNGase F deposition, tissue slides were carefully transferred to the pre-heated humid chamber and incubated overnight at 37 °C in an oven.

### 2.6. MALDI Matrix Deposition

The following day, three navigation points were marked in each tissue’s surrounding using a white Tipp-Ex, after which the slides were scanned at high resolution using a digital slide scanner (Reflecta MF 5000; reflecta, Eutingen im Gäu, Germany). Subsequently, 1 μL of DH standard was manually spotted on each slide within the tissue-free region. After drying, MALDI matrix solution (CHCA; 7 g/L in 70% ACN with 0.1% TFA) was deposited over the entire tissue slides using the HTX TM-Sprayer (HTX Technologies, LLC, Chapel Hill, NC, USA). The spraying parameters were set as follows: temperature, 77 °C; flow rate, 0.1 mL/min; and passes, 6.

### 2.7. MALDI-MSI Measurement

The MALDI-TOF-MS analysis was performed in positive ion reflectron mode using a rapiflex MALDI Tissuetyper system (Bruker Daltonics, Bremen, Germany), within a mass-to-charge (*m/z*) window of 1100–5000, a raster width of 50 μm, 500 laser shots per spot, and sampling rate of 1.25 GS/s. Acquisition of MSI data was enabled by the flexImaging 5.1 and flexControl 3.0 software (Bruker Daltonics, Bremen, Germany). Prior to the measurement, external calibration was performed using a DH standard.

### 2.8. Hematoxylin and Eosin Staining

Following the measurement, CHCA was removed by submerging the slides in 70% ethanol for 1 min. The slides were rinsed with MilliQ water and were incubated in a ready-to-use Mayer’s hematoxylin solution for 10 min. The excess of hematoxylin was washed away by rinsing the tissue with warm, running tap water for 10 min. The efficiency of the staining was verified using a standard transmitted light microscope. Subsequently, the hematoxylin-stained slides were rinsed with MilliQ water and were counterstained with 1–2% eosin solution for 5 min. Following the eosin staining, tissue slides were rinsed with MilliQ water and then were dehydrated in increasing concentration of ethanol (70%, 80%, 90%, 96%, 3 × 100%; 1 min each) and xylene (3 × 1 min). At the end, the hematoxylin and eosin (H&E)-stained tissues were air-tightly sealed using Eukitt medium.

### 2.9. Data Analysis

Statistical analysis and annotation of regions of interest (i.e., tumor, tumor-stroma) were performed in SCiLS Lab software, version 2019a Pro (Bruker Daltonics, Bremen, Germany). MALDI- TOF mass spectra were total ion current normalized and the detected peaks were revised manually. The annotation of the corresponding *N*-glycan structures was performed with the assistance of the GlycoWorkbench software [25], based on the detected *m/z* values, the knowledge of glycobiological processing, and the existing literature data [20,21,22]. Due to the exploratory character of this study, tissue cores characterized by a high degree of calcification, extensive necrosis, and/or fibrosis as well as those that were damaged markedly during tissue processing were excluded from the analysis. The final analysis included a collection of the best-preserved TMA cores, which corresponded to four cores of LGSOC, six cores of CCC, and seven cores of both EC and BOT. The H&E stainings of all tissue cores included in the analysis are presented in Supplementary Material Figure S1. For each tissue core, tumor and adjacent tumor-stroma regions were annotated by a reference pathologist. It is important to note that, since all TMA cores were originally extracted from tumor-containing regions of donor FFPE tissue specimens, regions annotated as tumor-stroma encompassed tumor-surrounding areas, which were not entirely tumor-free but contained distinctly less tumor cells as compared to solid tumor regions. The potential of the in situ detected *N*-glycan structures to discriminate these two morphologically different tissue regions was determined using ROC curve analyses, performed separately for each investigated EOC histotype. To correct for unequal tissue sizes, all ROC curve analyses were performed on a random subset of collected mass spectra, assuring that in each test the number of mass spectra was identical for both compared groups. An area under the curve (AUC) value of 0.65 was considered a cut-off value. The discriminative potential of *N*-glycan structures was classified as poor (0.65 ≤ AUC < 0.7), fairly good (0.7 ≤ AUC < 0.8), good (0.8 ≤ AUC < 0.9), very good (0.9 ≤ AUC < 0.95), and excellent (AUC ≥ 0.95).

## 3. Results

### 3.1. In Situ N-Glycosylation Profiling of EOC Tissues

**Figure 1:**
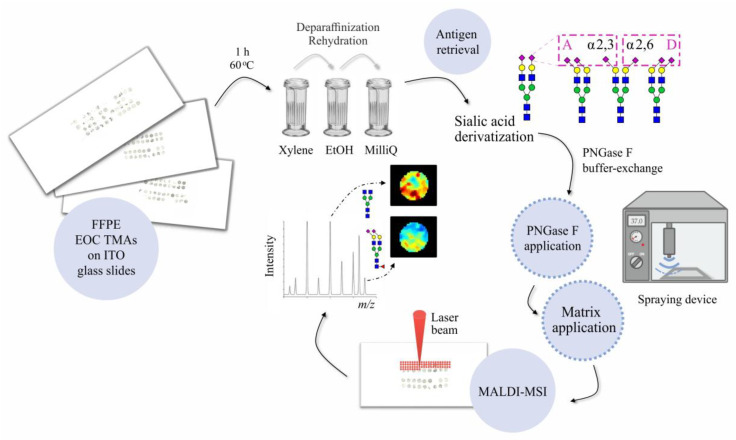
Schematic representation of the analytical workflow used in this study.

In this study, the MALDI-MSI technique was applied to determine tissue-specific *N*-glycosylation signatures of the less common EOC histotypes and non-malignant borderline ovarian tumor (BOT). After establishing the protocol and validating the regio-specificity of *N*-glycan profiles using the whole slide EOC tissue specimen (Supplementary Material Figure S2 and Figure S3), we performed the analysis of four TMA sections composed of tissue cores of LGSOC, CCC, EC, and BOT. The final analysis included a total of 24 tissue cores, whose clinicopathological characteristics are summarized in Supplementary Material Table S1. All investigated tissues were FFPE and were processed according to the procedure outlined in Figure 1.

As depicted in Figure 1, all TMA slides were initially incubated for 1 h at 60 °C in order to denature tissue-embedded proteins and enhance the adherence of tissue cores to ITO glass slides. Subsequently, each TMA section was deparaffinized, rehydrated, and antigen retrieved, after which it was subjected to a linkage-specific sialic acid derivatization, according to the protocol adapted from Holst et al. [21]. The schematic representation of the reaction mechanism together with its specificity determined using α2,3- and α2,6-sialylated standards as model samples is shown in Supplementary Material Figure S4. The derivatization reaction was important for two reasons. (1) It stabilizes labile sialic acids and prevents their in- and post-source decay. (2) Due to implementation of two linkage-specific amidation steps, it enables the differentiation of α2,3- and α2,6-sialic acids, hence, providing important biological information.

Following the chemical derivatization, tissue-residing *N*-glycans were released in situ using the PNGase F enzyme and, after MALDI matrix deposition, were measured by MALDI-TOF-MS. In parallel, to allow the identification of PNGase F-specific *m/z* signals, one OC tissue section served as a negative control and was processed omitting the PNGase F application step.

**Figure 2:**
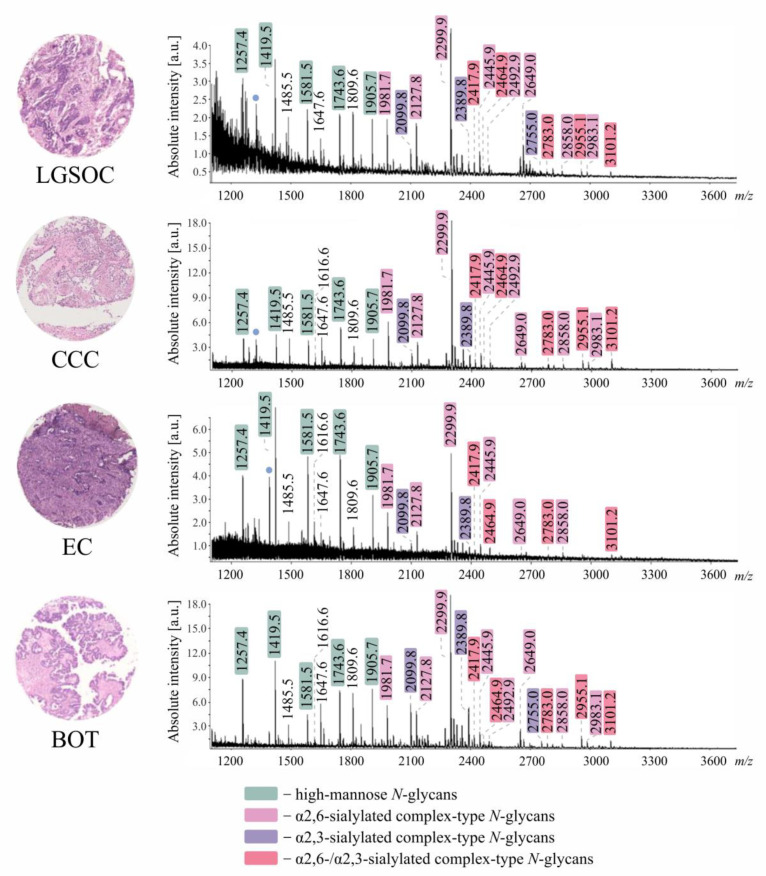
Average [M+Na]^+^ MALDI imaging mass spectra of all investigated tissue specimens (LGSOC *n* = 4, CCC *n* = 6, EC *n* = 7, BOT *n* = 7). *N*-glycan compositions of indicated *m/z* species are shown in Table 1. For each TMA slide, only one representative tissue core is shown. Blue dots: non-carbohydrate contaminants.

A varying number of *N*-glycan structures was detected across all investigated tissue specimens (Table 1), with over 50 *N*-glycan species being detected in BOT and less than 20 in EC. Assigned *N*-glycan structures corresponded to high-mannose, hybrid-, and complex-type neutral and sialylated *N*-glycans, frequently decorated with a fucose residue. Although, as visible in Figure 2, all investigated tissue specimens showed noticeably different average *N*-glycosylation profiles, in each case, recorded mass spectra were primarily dominated by *m/z* species corresponding to complex-type sialylated and high-mannose *N*-glycans.

**Figure 3:**
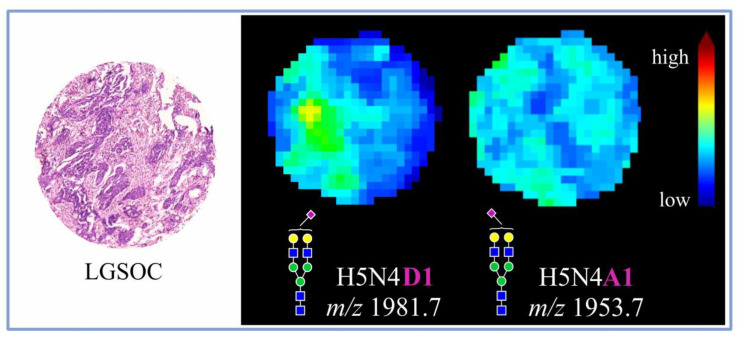
Spatial distribution of two differently sialylated *N*-glycan structures in an exemplary LGSOC tissue core as determined by MALDI-MSI.

Importantly, due to the application of chemical derivatization, it was possible to distinguish α2,3- from α2,6-sialylated *N*-glycan species, revealing the dominance of α2,6- over α2,3-sialylated forms. Most interestingly, α2,3- and α2,6-sialylated *N*-glycans were observed to exhibit distinct tissue distribution, as visualized in Figure 3 for an exemplary core of LGSOC-TMA specimen with a mono-α2,6-sialylated H5N4D1 structure at *m/z* 1981.7 and its α2,3-sialylated counterpart H5N4A1 at *m/z* 1953.7.

### 3.2. Discriminatory Power of In Situ Released N-Glycans in TMA Ovarian Cancer Specimens

**Figure 4:**
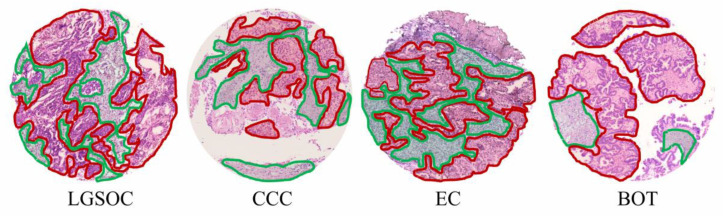
H&E stains of exemplary TMA cores investigated in this study. Tumor regions are marked in red, whereas tumor-stroma is marked in green.

Following the MALDI-MSI analysis, all investigated tissues were H&E stained, and morphological tissue regions corresponding to tumor and tumor-stroma were annotated by a pathologist, an example of which is shown in Figure 4.

Afterwards, receiver operating characteristic (ROC) curves were built for all *N*-glycan structures detected in investigated EOC and BOT tissue specimens (separately for each tissue histotype), in order to evaluate their utility as tissue type-specific determinants. Area under the curve (AUC) values for *N*-glycans exhibiting the highest discriminatory potential, as determined by tumor vs. tumor-stroma and tumor-stroma vs. tumor analyses, are shown in Table 2. Based on their corresponding AUC values, *N*-glycans were classified as poor (0.65 ≤ AUC < 0.7), fairly good (0.7 ≤ AUC < 0.8), good (0.8 ≤ AUC < 0.9), and very good (0.9 ≤ AUC < 0.95) discriminants of respective tissue regions.

As determined by the tumor vs. tumor-stroma ROC curve analyses, in the investigated tissue cohort, the best discrimination of tumor regions was achieved with α2,6-sialylated complex-type and high-mannose *N*-glycans. In the case of EC tissues, AUC ≥ 0.65 was recorded for eight *N*-glycan structures, of which five (i.e., H5N4D1 at *m/z* 1981.7, H5N5F1D1 at *m/z* 2330.9, H5N4F1D2 at *m/z* 2445.9, H5N4F1D1 at *m/z* 2127.8, and H4N4F1 at *m/z* 1647.6) displayed fairly good discriminative potential. In the case of OCC tissues, AUC ≥ 0.65 was recorded for one *N*-glycan structure, namely high-mannose H8N2 at *m/z* 1743.6 (AUC: 0.658). Contrarily, in LGSOC, no *N*-glycan showed AUC ≥ 0.65. Interestingly, the best results of the tumor vs. tumor-stroma ROC curve analysis were observed for BOT-TMA specimen. Consistent with the trends observed in EC and OCC EOC tissues, the best discriminators of BOT tumor regions were α2,6-sialylated complex- and high-mannose-type *N*-glycans. Among the α2,6-sialylated species, H5N5F1D1 at *m/z* 2330.9 showed good discriminative potential (AUC 0.806), whereas H5N5F1D2 at *m/z* 2649.0, H6N5D1 at *m/z* 2346.9, and H6N6F1D1 at *m/z* 2696.0 displayed fairly good discriminative potential. Similarly good results were achieved for high-mannose *N*-glycans, such as H9N2 at *m/z* 1905.6, H6N2 at *m/z* 1419.5, and H7N2 at *m/z* 1581.5.

As can be seen in the lower panel of Table 2, the following ROC curve analyses of tumor-stroma vs. tumor regions of LGSOC, CCC, EC, and BOT tissues indicated α2,3-sialylated *N*-glycans as the best determinants of tumor-stroma regions. Above all, H5N4F1A1 at *m/z* 2099.8 was revealed as a very good discriminant (AUC 0.901) of tumor-stroma in CCC and a fairly good discriminant in both EC and BOT specimens. In the case of LGSOC, no *N*-glycan showed AUC ≥ 0.65.

**Figure 5:**
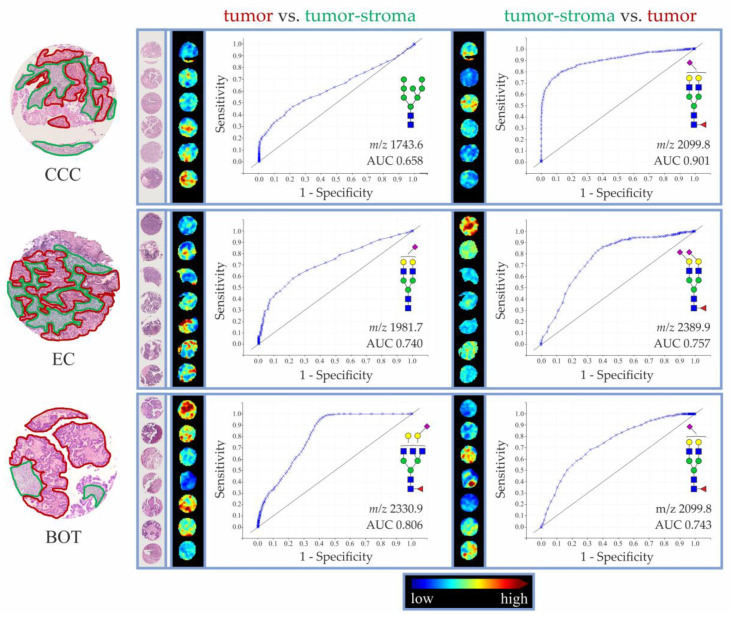
ROC curves and MALDI-MSI pictures of the most discriminatory *N*-glycan structures as determined for tumor and tumor-stroma in CCC (*n* = 6), EC (*n* = 7), and BOT (*n* = 7) TMA specimens. Annotation of tissue regions is shown only for one representative core of each histotype. Tumor regions are marked in red, whereas tumor-stroma is marked in green.

The ROC curves and the AUC values of the most discriminative *N*-glycan structures in CCC, EC, and BOT TMA specimens (tumor vs. tumor-stroma and tumor-stroma vs. tumor) are shown in Figure 5.

## 4. Discussion

The current knowledge on OC-related *N*-glycosylation changes has been predominantly gained from studies of glycoproteins contained in body fluids [8,9,26,27,28,29]. Throughout the years, this approach revealed a number of *N*-glycosylation alteration patterns that hold the potential for improving OC diagnosis, monitoring, and treatment. Nevertheless, as glycoproteins and *N*-glycans shed from tumor lesion represent only a minute fraction of all molecules contained in body fluids, glycosylation changes determined at the level of total serum/ plasma/ blood or ascites reflect primarily the systemic response of the host to the tumor rather than tumor glycosylation profile itself.

Therefore, in order to deepen the understanding of OC-related *N*-glycosylation changes, in this study, the analysis of enzymatically released *N*-glycans was executed directly on tissue, implementing the MALDI-MSI technique. By combining the high sensitivity of mass spectrometry with spatial distribution information typically obtained from immunostainings, this cutting-edge technique enables spatial profiling of numerous analytes in a single imaging experiment. Nevertheless, despite its obvious benefits, MALDI-MSI has so far been implemented in only two studies aiming at profiling *N*-glycosylation changes in OC tissue [20,22]. Importantly, as far as these studies provided solid information on the distribution of high-mannose and complex-type neutral *N*-glycans in OC tissue, they presented very limited data regarding sialylated *N*-glycan species. This shortcoming is most likely due to the poor detection of native sialylated structures in MALDI-TOF-MS operated in positive ionization mode.

To fill this knowledge gap, in this study, EOC tissue-specific *N*-glycosylation profiling was further expanded by introducing linkage-specific sialic acid derivatization [21], which significantly improved MALDI-MSI detection of sialylated *N*-glycans and enabled the discrimination of their α2,3- and α2,6-linkage to adjacent galactoses. Most importantly, implementation of the chemical derivatization allowed revealing distinct spatial distribution of α2,3- and α2,6-sialylated *N*-glycans that was associated with EOC tissue morphology. Specifically, the results of this work demonstrated that, besides high-mannose *N*-glycans, described already in previous studies [20,22], tumor regions of EOC tissues are marked by a high abundance of complex-type α2,6-sialylated *N*-glycans. In fact, the biantennary bi-α2,6-sialylated H5N4D2 structure represented one of the most abundant *N*-glycan structures across all investigated EOC tissues. In turn, adjacent tumor-stroma regions were observed to be enriched in complex-type α2,3-sialylated and neutral *N*-glycans.

As such, data presented in this work bring new information on the two-dimensional distribution of sialylated *N*-glycans within malignant and non-malignant OC tissues and extend the findings of Everest-Dass et al. [20] and Briggs et al. [22], who both concentrated on high-mannose and complex-type neutral *N*-glycans in OC tissues, and of Young et al., [30] who recently investigated early- versus late-stage serous OC tissues using PGC-LC-ESI-MS/MS, highlighting the importance of α2,3- and α2,6-sialylation in cancer.

Notably, our results are mostly in agreement with data reported by Anugraham et al., who investigated membrane *N*-glycosylation in four cancerous (SKOV 3, IGROV 1, A2780, and OVCAR 3) and two non-cancerous (HOSE 6.3 and HOSE 17.1) ovarian cell lines using PGC-LC-ESI-MS/MS [31]. Specifically, the authors of this study demonstrated that high-mannose *N*-glycans are more abundant in ovarian cancerous cell lines, whereas complex-type neutral structures are higher in non-cancerous cells. Furthermore, by implementing α2,3-sialidase treatment, Anugraham et al. [31] demonstrated that, in terms of sialylation, membranes of cancerous cells are predominantly decorated with α2,6-sialylated *N*-glycans. The latter results were further confirmed at the genetic level, as only cancerous cell lines showed a high expression of ST6GAL1, a gene encoding for sialyltransferase catalyzing the addition of α2,6-linked sialic acids. Besides increased high-mannosylation and the prevalence of α2,6-sialylation, OC cell line-specific *N*-glycosylation profiles were shown to be marked by the presence of specific bisecting and LacdiNAc-type *N*-glycans, with the latter representing less than 1% of total relative ion intensities. Contrarily, Zhang et al. [32] showed using metabolic stable isotope labeling that bisecting GlcNAc was reduced in the highly metastatic SKOV 3 OC cell line derivative (SKOV 3-ip) compared to low-metastatic SKOV 3, which highlights the link between cellular glycosylation and the metastatic phenotype. Since, as opposed to PGC-LC-ESI-MS/MS, the MALDI-TOF-MS device implemented in this work did not allow the enrichment and fragmentation of in situ detected analytes, the presence and distribution of these lowly abundant *N*-glycan species could not be validated in investigated EOC tissues. Nevertheless, since the results of Anugraham and coworkers [31] are largely in line with the MALDI-MSI data presented here, SKOV 3, IGROV 1, A2780, and OVCAR 3 OC cell lines seem to represent a quite reliable in vitro model of OC tissue-specific *N*-glycosylation. The *N*-glycome of OC tissues from patients was previously analyzed by Chen et al. [33] using MALDI-TOF/TOF-MS and a permethylation protocol to derivatize sialic acids. The authors reported an increase in high-mannose structures, which is consistent with our data, and a decrease in non-fucosylated complex-type structures.

It is important to note that EOC tissue-specific *N*-glycosylation profiles, marked by high-mannosylation and α2,6-sialylation, might have important functional and clinical implications. As shown by Park et al. [34], in cholangiocarcinoma (CCA), an epithelial neoplasm of bile ducts, the elevation of high-mannose *N*-glycans on the surface of cancer cells was associated with their enhanced metastatic spread. Notably, an increased abundance of high-mannose structures in CCA cells was shown to result from the diminished expression of α-1,2-mannosidase-coding genes, mainly MAN1A1, whose resulting protein product is responsible for trimming immature *N*-glycans prior to their processing to hybrid- and complex-type structures. In agreement with those data, forced overexpression of MAN1A1 in metastatic CCA cell lines hampered tumor growth *in vivo*. Furthermore, it was demonstrated that the migratory and invasive capabilities of metastatic CCA cells can be compromised by masking extracellular α1,2-mannosylation with a mannose-binding lectin [34]. It is, however, uncertain to what extent the above trend is valid for OC, as the analysis of over 200 surgical tissue specimens revealed that high MAN1A1 expression levels correlate with advanced OC stage, the presence of distant metastasis, and shorter relapse-free survival [35]. However, the subsequent analysis of three OC cell lines revealed a great variance in MAN1A1 expression levels, suggesting that the expression of the latter gene may be cell- and tissue type-specific [35]. Since, in our study, OC tumor regions were marked by increased abundance of high-mannose *N*-glycans, further research is necessary to more precisely define the molecular backgrounds and regulations of this glycosylation feature.

Aside from the increased abundance of high-mannose *N*-glycans, the EOC tissues investigated in this work were characterized by particularly high α2,6-sialylation, which might likewise have major clinical implications. Hypersialylation has long been associated with enhanced cell survival and metastatic behavior, the effects of which are elicited by diverse molecular mechanisms. For instance, increased α2,6-sialylation of the Fas death receptor was shown to impair Fas-mediated apoptosis, allowing cancer cells to evade a major mechanism of programmed cell death [36]. Similarly, as demonstrated in colon cancer cells, an increase in terminal α2,6-sialylation on β1-integrins blocks their binding to extracellular galectin-3, inhibiting galectin-3-mediated apoptosis [37]. Of note, resistance to apoptosis is crucial for cancer cell survival and tumor progression; for that reason, it is classified as a hallmark of cancer [38]. Interestingly, the above-described inhibition of galectin-3 binding was shown to occur specifically in the presence of α2,6- and not α2,3-sialylated oligosaccharides [39], which further emphasizes the importance of linkage-specific examination of sialylated glycoforms in cancer tissues. Furthermore, as demonstrated in OV4 ovarian cancer cells that lack endogenous sialyltransferase activity, forced expression of ST6Gal-I resulted in α2,6-sialylation of β1-integrin, leading to reduced cell–cell adherence and increased motility of affected cells [40]. A similar effect of forced ST6Gal-I expression was described in breast carcinoma cells [41], which further confirms the notion that increased α2,6-sialylation associates with an invasive phenotype. Most importantly, Schultz et al. [42] showed that upregulated endogenous or forced expression of ST6Gal-I in OC cell lines is linked to cisplatin resistance. The latter finding indicates that efficiency of OC treatment, which, besides operative debulking, typically consists of platinum-based chemotherapy, is, among other factors, dependent on cancer cells’ sialylation status.

It should be noted that the presented proof-of-concept study suffers from some limitations. First and foremost, the analyses were conducted on a small tissue cohort; for that reason, the presented *N*-glycosylation profiles require validation in a bigger and more diversified tissue set. In particular, it would be of interest to determine whether in situ sialylation of EOC tissues differs with respect to FIGO stage and whether it alters upon treatment. Certainly, the presented work would benefit from implementation of other analytical techniques, for instance laser capture microdissection (LCM) that enables precise isolation of distinct subpopulations of tissue-contained cells under direct microscopic visualization [43]. Specifically, LCM coupled with high- and/or ultra-resolution mass spectrometry could allow more comprehensive *N*-glycoprofiling of tumor and tumor-stroma regions of investigated EOC lesions, facilitating the discovery of tissue type-specific glyco-biomarkers. Nonetheless, as LCM-based cell fractionation might be time-consuming, its applicability for high-throughput studies is limited. Finally, understanding of the observed EOC tissue-specific *N*-glycosylation profiles and their potential implications for OC progression and treatment could be complemented by the identification of the corresponding glycan carrier proteins. Admittedly, consecutive slides of TMA specimens analyzed in this work constituted a part of the EOC tissue material investigated in the MALDI-MSI proteomic study, in which data were processed using various machine learning methods to discriminate histologic EOC subtypes [44]. The co-registration of glycomics’ data generated in this study and peptidomics’ data reported by Klein et al. was, however, not possible due to small number of TMA cores giving good-quality *N*-glycan signals. Hence, further analyses are necessary in order to define protein carriers of tumor-contained high-mannose and α2,6-sialylated *N*-glycans as well as tumor-stroma-contained α2,3-sialylated *N*-glycans in OC tissue.

## 5. Conclusions

In this study, we implemented MALDI-MSI in combination with a chemical derivatization reaction reported by Holst et al. [21] to simultaneously delineate the in situ spatial distribution of neutral and sialylated *N*-glycan species in EOC tissue specimens. Although our data necessitate a validation using a larger cohort, they provide a valuable insight into molecular changes occurring directly within EOC tissue. In fact, MALDI-MSI profiling revealed that, besides high-mannose *N*-glycans, EOC tissues are abundantly decorated with sialylated glycan structures. Most importantly, presented data indicate that α2,6- and α2,3-sialylated *N*-glycan structures differ with respect to in situ EOC tissue distribution, highlighting the importance of the linkage-specific sialic acid analysis. All in all, we demonstrated that the combination of MALDI-MSI and sialic acid derivatization is suitable for delineating regio-specific *N*-glycan distribution in EOC and BOT tissues and might serve as a promising strategy for future glycosylation-based biomarker discovery studies.

## Figures and Tables

**Table 1:** *N*-Glycan structures detected by MALDI-MSI across all investigated tissue specimens (LGSOC *n* = 4, CCC *n* = 6, EC *n* = 7, BOT *n* = 7).

*m/z* [M + Na]^+^	Composition	Neg	LGSOC	CCC	EC	BOT
1136.40	H3N3					
1257.42	H5N2		✓	✓	✓	✓
1282.45	H3N3F1					
1298.45	H4N3					
1339.48	H3N4					
1419.48	H6N2		✓	✓	✓	✓
1444.51	H4N3F1					✓
1485.53	H3N4F1		✓	✓	✓	✓
1501.53	H4N4		✓			✓
1581.53	H7N2		✓	✓	✓	✓
1616.60	H4N3D1			✓		✓
1647.59	H4N4F1		✓	✓		✓
1663.58	H5N4		✓	✓		✓
1688.61	H3N5F1					✓
1704.61	H4N5					✓
1743.58	H8N2		✓	✓	✓	✓
1762.65	H4N3F1D1					✓
1778.64	H5N3D1					✓
1809.64	H5N4F1		✓	✓	✓	✓
1819.68	H4N4D1					✓
1825.63	H6N4					✓
1850.67	H4N5F1		✓	✓		✓
1866.66	H5N5					✓
1891.69	H3N6F1					
1905.63	H9N2		✓	✓	✓	✓
1937.70	H4N4F1A1					
1953.70	H5N4A1		✓	✓		✓
1965.73	H4N4F1D1		✓			✓
1981.73	H5N4D1		✓	✓	✓	✓
2012.72	H5N5F1		✓	✓	✓	✓
2028.71	H6N5					✓
2053.75	H4N6F1					
2099.76	H5N4F1A1		✓	✓	✓	✓
2127.79	H5N4F1D1		✓	✓	✓	✓
2168.82	H4N5F1D1		✓			✓
2174.77	H6N5F1		✓			✓
2184.80	H5N5D1		✓	✓		✓
2243.81	H5N4A2					✓
2271.85	H5N4A1D1		✓	✓		✓
2299.88	H5N4D2		✓	✓	✓	✓
2330.87	H5N5F1D1		✓	✓	✓	✓
2346.86	H6N5D1		✓	✓		✓
2389.88	H5N4F1A2		✓	✓	✓	✓
2417.91	H5N4F1A1D1		✓	✓		✓
2445.94	H5N4F1D2		✓	✓	✓	✓
2464.89	H6N5F1A1		✓	✓		✓
2492.91	H6N5F1D1		✓	✓	✓	✓
2502.95	H5N5D2		✓	✓		✓
2539.90	H7N6F1					
2636.97	H6N5A1D1			✓		✓
2649.00	H5N5F1D2		✓	✓		✓
2665.00	H6N5D2			✓		✓
2696.00	H6N6F1D1		✓	✓		✓
2755.00	H6N5F1A2			✓		✓
2783.03	H6N5F1A1D1		✓	✓		✓
2811.06	H6N5F1D2		✓	✓		✓
2830.01	H7N6F1A1					
2858.05	H7N6F1D1		✓	✓	✓	✓
2955.11	H6N5A1D2		✓	✓		✓
2983.14	H6N5D3		✓	✓		✓
3045.12	H6N5F1A3			✓		✓
3073.14	H6N5F1A2D1			✓		✓
3101.17	H6N5F1A1D2		✓	✓	✓	✓
3148.16	H7N6F1A1D1			✓		✓
3158.19	H6N6A1D2			✓		✓

Neg–negative control; ✓—detected peak. *N*-Glycan composition is given in terms of H, hexose; N, *N*-acetylhexosamine; F, fucose; A, amidated sialic acid; and D, dimethylamidated sialic acid.

**Table 2:** The most discriminative *N*-glycan structures as determined by ROC curve analyses of tumor vs. tumor-stroma and tumor-stroma vs. tumor regions in LGSOC (*n* = 4), CCC (*n* = 6), EC (*n* = 7), and BOT (*n* = 7) tissue specimens.

Tumor vs. Tumor-stroma											
LGSOC			CCC			EC			BOT		
*m/z*	Composition	AUC	*m/z*	Composition	AUC	*m/z*	Composition	AUC	*m/z*	Composition	AUC
-	-	-	1743.6	H8N2	0.658	1981.7	H5N4D1	0.740	2330.9	H5N5F1D1	0.806
						2330.9	H5N5F1D1	0.735	2649.0	H5N5F1D2	0.775
						2445.9	H5N4F1D2	0.728	1905.6	H9N2	0.759
						2127.8	H5N4F1D1	0.727	1419.5	H6N2	0.758
						1647.6	H4N4F1	0.722	2346.9	H6N5D1	0.732
						2492.9	H6N5F1D1	0.682	2696.0	H6N6F1D1	0.724
						1419.5	H6N2	0.670	1581.5	H7N2	0.719
						1581.5	H7N2	0.667	3101.2	H6N5F1A1D2	0.700
Tumor-stroma vs. Tumor											
LGSOC			CCC			EC			BOT		
*m/z*	Composition	AUC	*m/z*	Composition	AUC	*m/z*	Composition	AUC	*m/z*	Composition	AUC
-	-	-	2099.8	H5N4F1A1	0.901	2389.9	H5N4F1A2	0.757	2099.8	H5N4F1A1	0.743
			1953.7	H5N4A1	0.825	2099.8	H5N4A1	0.734	1809.6	H5N4F1	0.670
			1981.7	H5N4D1	0.822				2012.7	H5N5F1	0.662
			2955.1	H6N5A1D2	0.815						
			2665.0	H6N5D2	0.774						

Only *N*-glycan structures with AUC > 0.65 are shown. In the case of EC and BOT, eight *N*-glycan structures with the highest AUC values are shown.

## Data Availability

Data are contained within the article or Supplementary Materials. The MALDI-MSI data presented in this study are available on request from the corresponding author.

## Supplementary Materials

The following are available online at https://www.mdpi.com/article/10.3390/cancers14041021/s1. Figure S1: H&E stains of TMA cores included in the MALDI-MSI analysis. Figure S2: Spatial distribution of exemplary *N*-glycan structures in EOC whole tissue section as determined by MALDI-MSI. Figure S3: Validation of *N*-glycan regio-specificity in EOC whole tissue specimen by off-tissue MALDI-TOF-MS analysis of *N*-glycan surface liquid extracts [45]. Figure S4: Linkage-specific sialic acid derivatization. Table S1: Clinicopathological characteristics of the investigated tissue cohort.
