# Supplementary material for: In Situ N-Glycosylation Signatures of Epithelial Ovarian Cancer Tissue as Defined by MALDI Mass Spectrometry Imaging
Source: Cancers (Basel). 2022 Feb 17;14(4):1021. doi: 10.3390/cancers14041021 (PMC8870006; doi:10.3390/cancers14041021)
Supplement: Supplementary file 1 [file cancers-14-01021-s001.zip › cancers-1548687-SI.pdf]

cancers

MDPI

Article

In Situ *N*-glycosylation Signatures of Epithelial Ovarian Cancer Tissue as Defined by MALDI Mass Spectrometry Imaging

Marta Grzeski, Eliane T. Taube, Elena I. Braicu, Jalid Sehouli, Véronique Blanchard and Oliver Klein

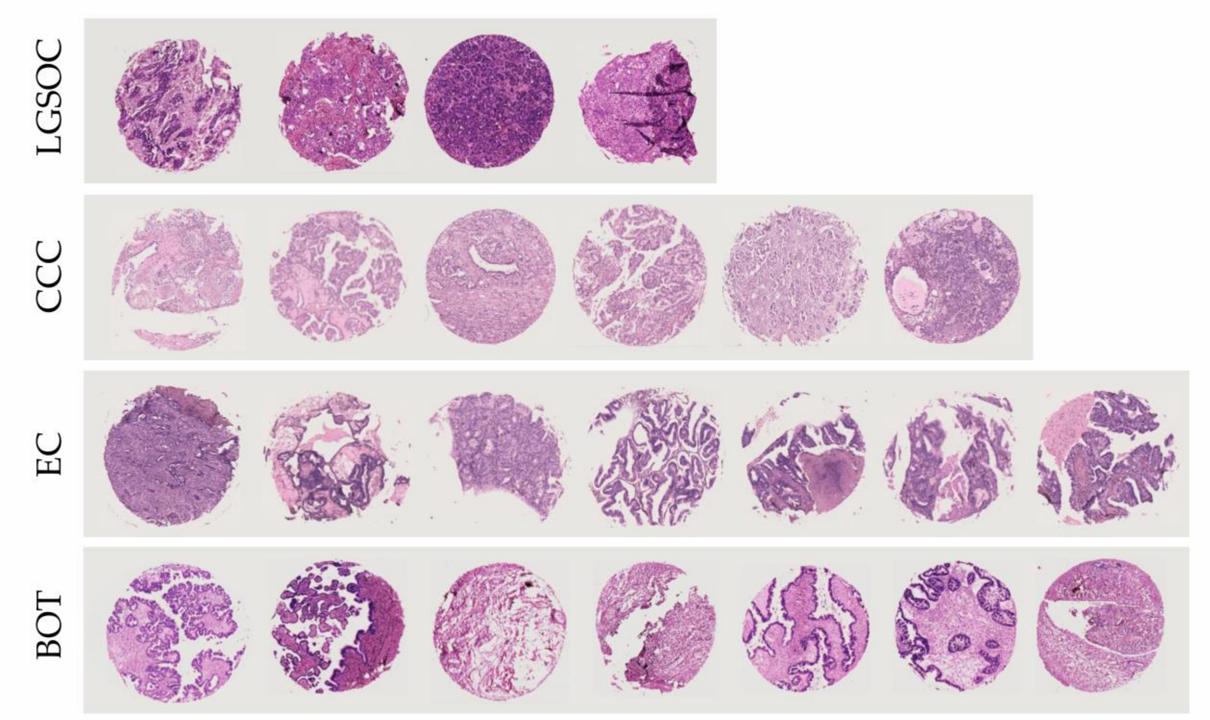

Figure S1. H&E stains of TMA cores included in the MALDI-MSI analysis.

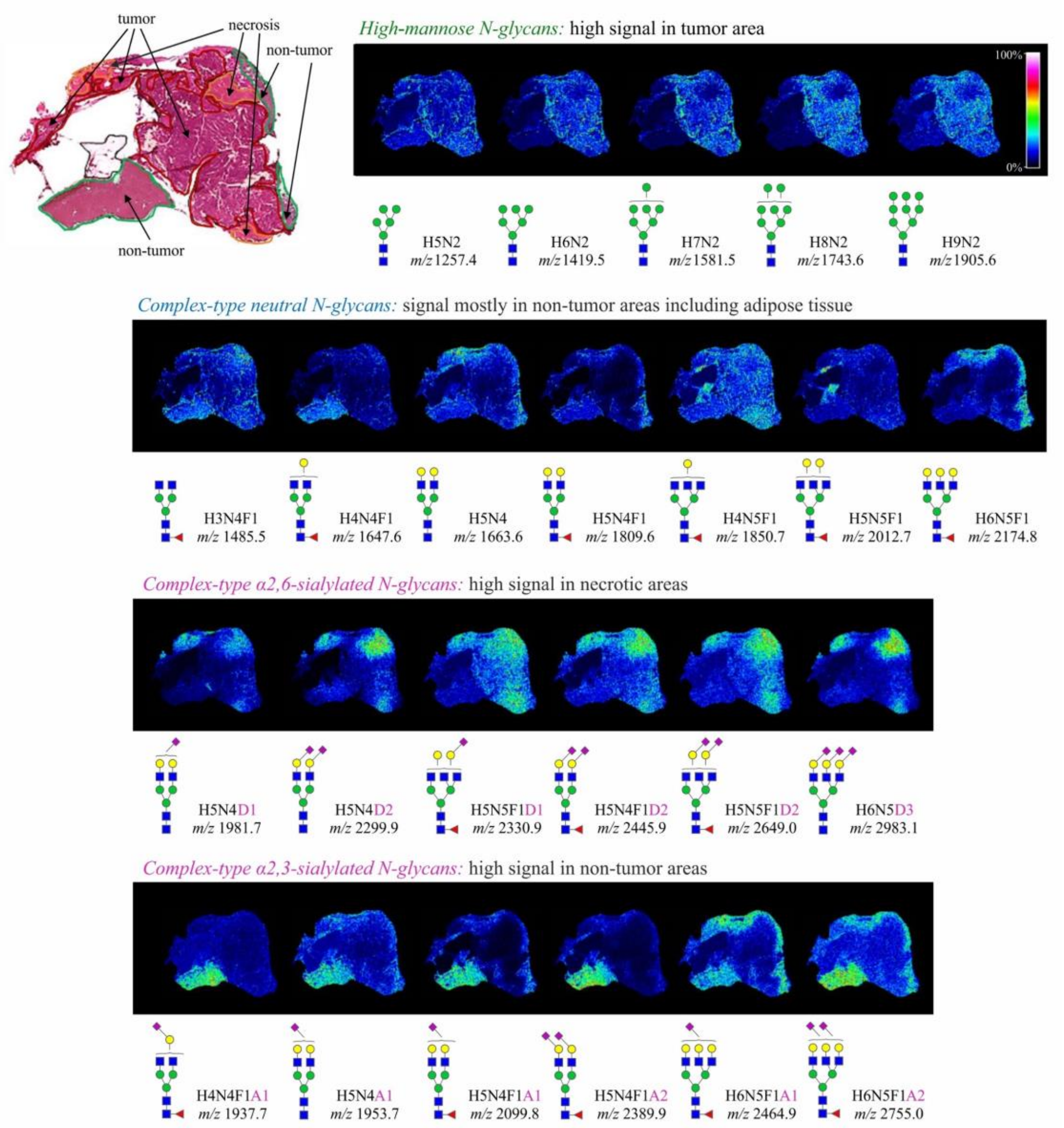

Figure S2. Spatial distribution of exemplary *N*-glycan structures in an EOC whole tissue section as determined by MALDI-MSI.

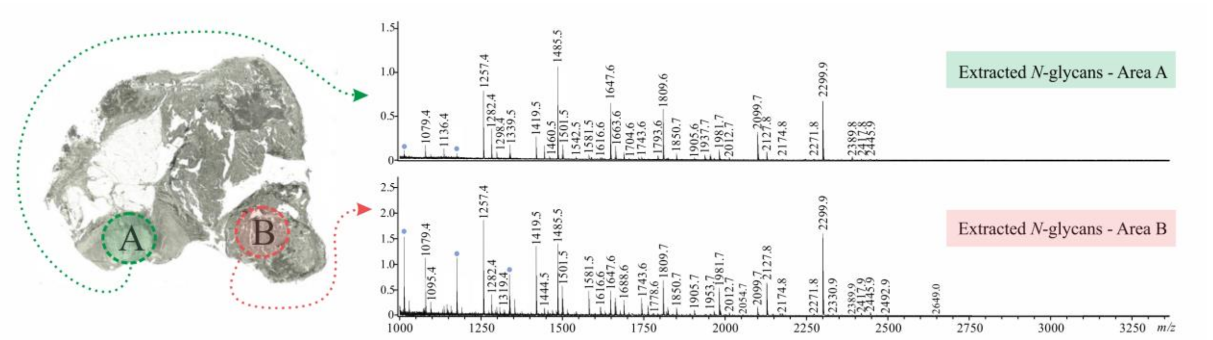

Figure S3. Validation of *N*-glycan regio-specificity in EOC whole tissue specimen by off-tissue MALDI-TOF-MS analysis of *N*-glycan surface liquid extracts.

The analysis was performed as indicated in the materials and methods section with small modification. Briefly, the FFPE EOC tissue specimen was deparaffinized, rehydrated, antigen retrieved, and subjected to chemical derivatization in order to stabilize terminal sialic acid residues (see Figure S4). Then, instead of depositing the enzyme with the spraying device, the buffer-changed PNGase F solution (2×10 µl) was carefully pipetted over two separate tissue parts, representing non-tumor (region A, marked in green) and tumor (region B, marked in red) regions, annotated by an experienced pathologist (see Figure S2). Following overnight incubation at 37 °C in a humid chamber, released *N*-glycans were extracted with MilliQ water, concentrated, and purified using self-made cotton-HILIC columns, as described by Selman et al. [45] . Eventually, each extracted *N*-glycan fraction was measured separately by MALDI-TOF-MS in positive ionization mode using super-DHB as MALDI matrix. As visible in the presented mass spectra, investigated tissue regions showed distinct *N*-glycosylation profiles. *N*-Glycan annotations of indicated *m*/*z* species are shown in Table 1. Blue dots: non-carbohydrate contaminants.

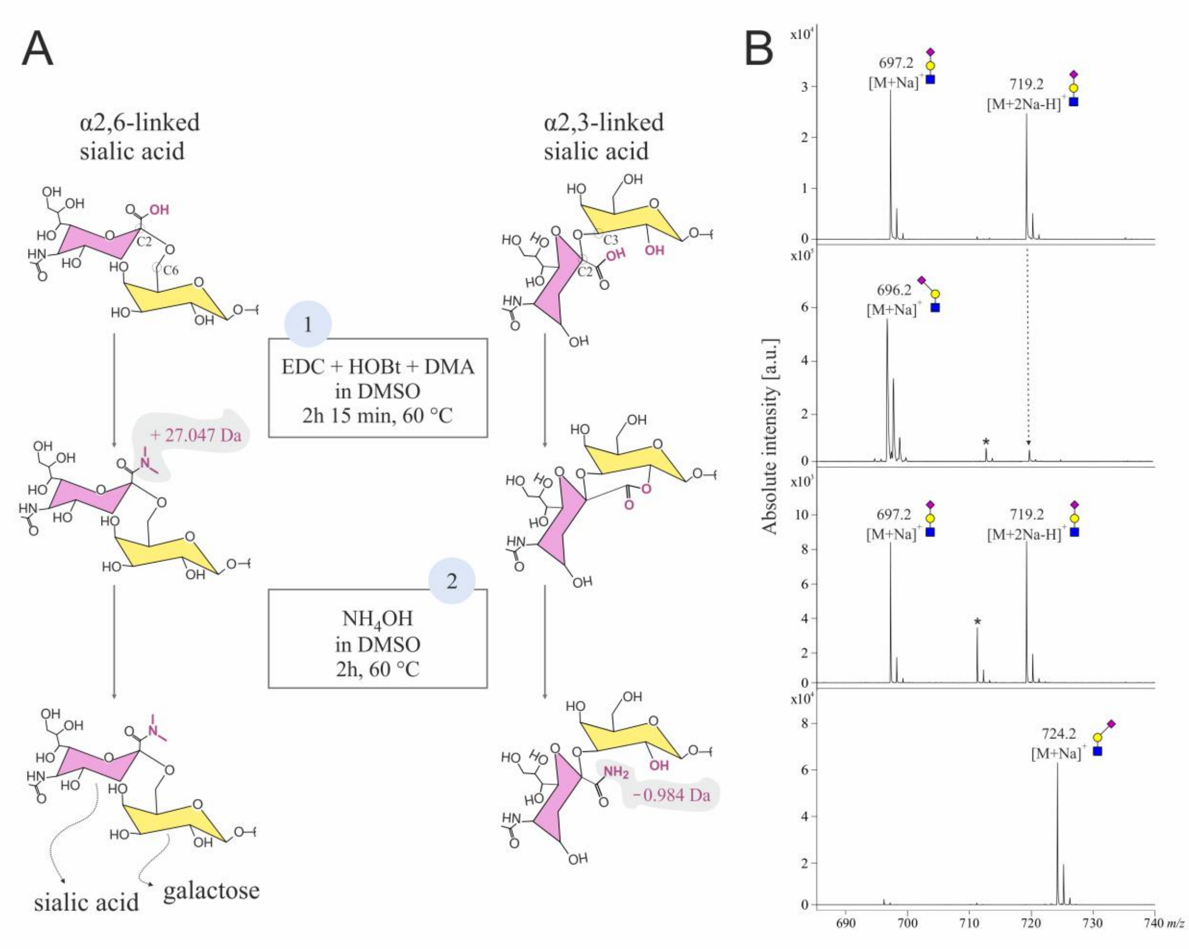

Figure S4. Linkage-specific sialic acid derivatization adapted from Holst et al. [21]. (a) Schematic representation of the reaction mechanism. Sialic acid is marked in pink, whereas galactose is marked in yellow. (b) MALDI-TOF mass spectra of non-derivatized and derivatized 3′- and 6′-SialLacNAc standards. From top to bottom: underivatized 3′- SialLacNAc, derivatized 3′-SialLacNAc, underivatized 6′-SialLacNAc, derivatized 6′-SialLacNAc. Monosaccharides are represented as follows: blue square, GlcNAc; yellow circle, Gal; pink diamond, Sia. Sialic acid leaning to the left and right represents α2,3- and α2,6-linkage, respectively. * unidentified peak.

Table S1. Clinicopathological characteristics of the investigated tissue cohort.

	Study group	LGSC	OEC	OCC	BOT
total n	24	4	7	6	7
age					
≤ 60 years	17 (70.8%)	3 (75%)	4 (57.1%)	4 (66.6%)	6 (85.7%)
> 60 years	7 (29.2%)	1 (25%)	3 (42.9%)	2 (33.3%)	1 (14.3%)
pT					
pT1	9 (52.9%)	-	4 (57.1%)	5 (83.3%)	
pT2	-	-	-	-	
pT3	6 (35.3%)	3 (75%)	2 (28.6%)	1 (16.6%)	
not determined	2 (11.8%)	1 (25%)	1 (14.3%)	-	
pN					
pN0	5 (29.4%)	-	1 (14.3%)	4 (66.6%)	
pN1	5 (29.4%)	2 (50%)	2 (28.6%)	1 (16.6%)	
pNx	7 (41.2%)	2 (50%)	4 (57.1%)	1 (16.6%)	
FIGO stage					
FIGO I	9 (52.9%)	-	4 (57.1%)	5 (83.3%)	
FIGO II	-	-	-	-	
FIGO III	5 (29.5%)	2 (50%)	2 (28.6%)	1 (16.6%)	
FIGO IV	1 (5.8%)	1 (25%)	-	-	
not determined	2 (11.8%)	1 (25%)	1 (14.3%)	-	
residual tumor					
0 cm	7 (41,2%)	2 (50%)	2 (28.6%)	3 (50%)	
> 0 cm	1 (5.8%)	1 (25%)	-	-	
not determined (FIGO I)	5 (29.5%)	-	3 (42.9%)	2 (33.3%)	
not determined	4 (23.5%)	1 (25%)	2 (28.6%)	1 (16.6%)	

pT, size or extend of primary tumor; pN, degree of spread to regional lymph nodes
